# Joint association between birth weight at term and later life adherence to a healthy lifestyle with risk of hypertension: a prospective cohort study

**DOI:** 10.1186/s12916-015-0409-1

**Published:** 2015-07-31

**Authors:** Yanping Li, Sylvia H. Ley, Tyler J. VanderWeele, Gary C. Curhan, Janet W. Rich-Edwards, Walter C. Willett, John P. Forman, Frank B. Hu, Lu Qi

**Affiliations:** Department of Nutrition, Harvard School of Public Health, Boston, MA USA; Department of Epidemiology, Harvard School of Public Health, Boston, MA USA; Department of Biostatistics, Harvard School of Public Health, Boston, MA USA; Channing Division of Network Medicine, Department of Medicine, Brigham and Women’s Hospital, Harvard Medical School, 665 Huntington Ave, Boston, MA 02115 USA; Renal Division, Department of Medicine, Brigham and Women’s Hospital, Boston, MA USA; The Connors Center for Women’s Health and Gender Biology, Brigham and Women’s Hospital, Harvard Medical School, Boston, MA USA

**Keywords:** Hypertension, Lifestyle, Nutrition, Women

## Abstract

**Background:**

Low birth weight and unhealthy lifestyles in adulthood have been independently associated with an elevated risk of hypertension. However, no study has examined the joint effects of these factors on incidence of hypertension.

**Methods:**

We followed 52,114 women from the Nurses’ Health Study II without hypercholesterolemia, diabetes, cardiovascular disease, cancer, prehypertension, and hypertension at baseline (1991–2011). Women born preterm, of a multiple pregnancy, or who were missing birth weight data were excluded. Unhealthy adulthood lifestyle was defined by compiling status scores of body mass index, physical activity, alcohol consumption, the Dietary Approaches to Stop Hypertension diet, and the use of non-narcotic analgesics.

**Results:**

We documented 12,588 incident cases of hypertension during 20 years of follow-up. The risk of hypertension associated with a combination of low birth weight at term and unhealthy lifestyle factors (RR, 1.95; 95 % CI, 1.83–2.07) was more than the addition of the risk associated with each individual factor, indicating a significant interaction on an additive scale (*P*_interaction_ <0.001). The proportions of the association attributable to lower term birth weight alone, unhealthy lifestyle alone, and their joint effect were 23.9 % (95 % CI, 16.6–31.2), 63.7 % (95 % CI, 60.4–66.9), and 12.5 % (95 % CI, 9.87–15.0), respectively. The population-attributable-risk for the combined adulthood unhealthy lifestyle and low birth weight at term was 66.3 % (95 % CI, 56.9–74.0).

**Conclusion:**

The majority of cases of hypertension could be prevented by the adoption of a healthier lifestyle, though some cases may depend on simultaneous improvement of both prenatal and postnatal factors.

## Background

Hypertension affects one third of American adults [1] and is the leading cause of global disease burden [2, 3]. Prenatal factors, such as intrauterine nutrition status [4–11], and adulthood factors, such as unhealthy diet and lifestyle [12, 13], have been independently associated with an elevated risk of hypertension. The association between prenatal famine exposure and high blood pressure was consistently observed in studies of the Dutch famine [11], the Leningrad Siege [10], and the Chinese famine [8]. In addition, low birth weight has been consistently associated with an increased risk of hypertension [4–6]. Compelling evidence has also related adulthood lifestyle such as body weight, diet, physical activity, and alcohol consumption with the development of hypertension; lifestyle modifications have been recommended for prevention of hypertension [12, 13].

However, no previous study has examined the joint associations of birth weight and adulthood lifestyle with hypertension risk, or evaluated their potential interactions. In this study, we prospectively assessed the joint association of birth weight at term – a marker of fetal growth restriction – and established lifestyle risk factors in adulthood with incident hypertension in the Nurses’ Health Study (NHS) II [14].

## Methods

### Study population

In 1989, 116,430 female registered nurses aged 25 to 42 years enrolled in NHS II by completing and returning an initial questionnaire that provided detailed information on medical history, lifestyle, and medications. The dietary survey was initiated using a food frequency questionnaire (FFQ) in 1991, which served as the baseline of the present analysis. Detailed information on lifestyle habits and medical history was updated biennially; FFQ was updated every 4 years. The follow-up for the cohort exceeded 90 % of eligible person-time.

In 1991, 100,090 participants returned the questionnaire including a FFQ. For the current analysis, we excluded participants (1) who reported diagnosis of hypertension, ever use of antihypertensive medication, or who reported median systolic blood pressure in the prehypertensive range, greater than 120 mmHg or diastolic blood pressure greater than 80 mm Hg, at or before baseline of the current analysis (1991, n = 18,468); (2) who reported physician diagnosis of other chronic conditions, including hypercholesterolemia, diabetes, cardiovascular disease, and cancer at baseline (n = 12,901); (3) who had missing data on dietary, physical activity, alcohol consumption, use of non-narcotic analgesics, or body weight at baseline (n = 6,904); and (4) who were born preterm (n = 4,339, defined as ‘born 2+ weeks premature’), did not provide birth weight data (n = 4,677), or who were born of a multiple pregnancy (n = 687). After these exclusions, 52,114 women were included in the analysis. Participants who did not report birth weight or missed lifestyle factors had similar age (mean 36.3 ± 4.7 vs 36.0 ± 4.7 years) and body mass index (BMI; 24.9 ± 5.5 vs 24.6 ± 5.3 kg/m^2^) as those with relative information.

The Institutional Review Boards at the Harvard School of Public Health and Brigham and Women’s Hospital approved the study protocol. The completion of the self-administered questionnaire was considered to imply informed consent.

### Ascertainment of hypertension

The baseline and follow-up biennial questionnaires asked participants to report whether a clinician had made a new diagnosis of hypertension during the preceding 2 years [14]. Self-reported hypertension was validated in a subset of this cohort using medical record review [15]. Of 51 women who reported hypertension and for whom records of blood pressure were available, the initial report was confirmed in all cases (blood pressure >140/90 mmHg). In a second survey, blood pressure was measured in a sample of Boston-area participants who were part of the diet validation study. Among the 161 participants sampled who did not report high blood pressure, none had a blood pressure greater than 160/95 mmHg and 6.8 % had values between 140/90 and 160/95 mmHg. This confirms a low rate of false negative reporting. Self-reported blood pressure and hypertension are also strong predictors of coronary heart disease in the NHS study [16]. Incident hypertension cases included individuals who first reported hypertension on questionnaires after 1991 [15].

### Ascertainment of birth weight

Participants in NHS II were asked about their birth weight on the 1991 questionnaires [14]. Five categories of birth weight responses (in lb) were specified: <5.5, 5.5–6.9, 7.0–8.4, 8.5–9.9, ≥10.0 (in kg: <2.5, 2.5–3.15, 3.16–3.82, 3.83–4.49, ≥4.5). A validation study on birth weight was reported previously [17]. In brief, the mean values (in lb) for the five birth weight categories calculated with the state birth records of 220 randomly selected NHS II participants were 4.8, 6.3, 7.6, 8.9, and 10.3 [17]. In addition, 70.0 % of the NHS II participants reported the same birth weight category as was obtained from state birth records [17]. The Spearman correlation between self-reported birth weight and weights recorded on state birth records was 0.74 (*P* <0.001) [17].

### Definition of unhealthy and healthy lifestyle

Five lifestyle factors were included in our healthy lifestyle score, namely diet, physical activity, alcohol consumption, use of non-narcotic analgesics, and BMI, based on the strength of evidence related to risk of hypertension [12]. For each lifestyle factor the participant received 1 if she met the criteria for low risk, and 0 otherwise. This lifestyle score predicted risk of hypertension in our previous study in this cohort [12]. In sensitivity analysis, we also assigned weights to each low-risk factor based on the beta coefficients from the multivariable-adjusted Cox model with incident hypertension as the outcome. We then summed up the products, divided it by the sum of all beta coefficient values, and then multiplied by 5 to make the low-risk lifestyle score easier to interpret, e.g. each unit of the expanded low-risk lifestyle score presented one risk factor.

For physical activity, we classified low risk as ≥3.5 hours/week of moderate or vigorous activity. We defined moderate alcohol consumption as greater than zero but not exceeding 10 g/d (approximately 1 alcohol beverage per day) for moderate alcohol intake. Low risk BMI was defined as BMI <25 kg/m^2^. We calculated the dietary score of the Dietary Approaches to Stop Hypertension (DASH) diet, which has been associated with blood pressure [18, 19]. Women with DASH scores in the top quintile (20 %) were classified as having a low-risk diet. The low-risk category of non-narcotic analgesic use was defined as the use that was less frequent than once per week, as previous studies have documented increases in the risk of incident hypertension with even a low frequency of non-narcotic analgesic use [20–22].

### Statistical analysis

We presented the baseline characteristics of the study population according to the category of term birth weight in Table 1. Values were means ± standard deviation or percentages and were standardized to the age distribution of the study population.

**Table 1:** Age-adjusted characteristics of participants according to term birth weight category at baseline (1991)

	Term birth weight categories (kg)				
	<2.5	2.5–3.15	3.16–3.82	3.83–4.49	≥4.5
N (52,114)	1,763	15,346	27,253	7,064	688
Percentage, %	3.4	29.4	52.3	13.6	1.3
Age, years*	36.4 ± 4.6	35.6 ± 4.6	35.6 ± 4.6	35.1 ± 4.6	35.7 ± 4.5
Body mass index, kg/m^2^	23.3 ± 4.2	23.4 ± 4.3	23.6 ± 4.2	24.0 ± 4.4	24.6 ± 4.8
Total energy intake, kcal/d	1791 ± 565	1779 ± 746	1800 ± 539	1806 ± 543	1796 ± 558
DASH score	23.5 ± 5.1	23.7 ± 5.1	23.9 ± 5.1	24.0 ± 5.1	24.1 ± 5.3
Alcohol intake, g/d	2.9 ± 5.4	3.2 ± 5.9	3.2 ± 5.8	3.0 ± 5.9	2.9 ± 5.2
Current smoking, %	12.3	11.3	11.1	11.3	9.5
Moderate/vigorous intensity exercise, h/wk	2.5 ± 3.8	2.5 ± 3.9	2.5 ± 3.9	2.5 ± 3.8	2.8 ± 4.8
Family history of hypertension, %	51.5	48.5	47.7	49.3	54.7
White, %	93.5	93.2	95.5	96.0	94.3
Use of oral contraceptive pills, %	12.5	11.2	11.2	11.3	12.1
Use of supplemental folic acid, %	41.6	43.0	43.3	42.9	44.1
Supplemental folic acid intake, μg/d	147 ± 239	160 ± 257	161 ± 258	166 ± 263	161 ± 248
Nonnarcotic analgesic use at least once per week, %					
Aspirin or aspirin-containing products	10.7	10.2	9.7	9.4	11.0
Ibuprofen	17.9	16.9	16.4	16.3	17.7
Acetaminophen	22.7	19.9	19.3	19.2	22.5

Values are means ± standard deviation (SD) or percentages and are standardized to the age distribution of the study population

*Value is not age adjusted

*DASH* Dietary Approaches to Stop Hypertension

Individuals contributed person-time from the return of the baseline questionnaire (1991) until the date of diagnosis of hypertension, diabetes, cardiovascular disease or cancer, death, loss to follow-up, or the end of the follow-up period (June 30, 2011), whichever came first.

Multivariable Cox proportional hazards models were used to estimate relative risk (RR) and 95 % confidence intervals (CIs) for the association between birth weight at term and hypertension risk, with participants in the middle category of birth weight at term (3.16–3.82 kg) as the reference group. A linear trend across birth weight categories was evaluated with a Wald test for linear trend by assigning the median value to each category and modelling this variable as a continuous variable. We adjusted for *a priori* potential confounders including age, ethnicity, a family history of hypertension, smoking status, supplemental folic acid intake, and oral contraceptive use. We also adjusted the lifestyle factors including alcohol consumption, physical activity, DASH score, and the use of non-narcotic analgesics for the association between birth weight at term and hypertension. In a secondary analysis, we also further adjusted for adult BMI. We ran separate models with and without BMI because BMI may be confounder or mediator of the association between birth weight and hypertension. We updated information during follow-up period by using the most recently available information.

Participants were also classified according to the joint categories of birth weight at term and the number of unhealthy lifestyle factors. Lifestyle factors were updated at each questionnaire cycle to reflect the most recent information as a time-varying variable. If data were missing at a given time point, data from the previous cycle was used. We defined the group with birth weight at term <2.5 kg and with five unhealthy lifestyle scores as the reference group (with the highest risk of hypertension) and used multivariable Cox proportional hazards models to estimate RRs. We evaluated whether the associations between birth weight at term and hypertension differed by adulthood lifestyle on both multiplicative and additive scales [23–25]. The multiplicative interaction was tested by comparing the –2 log likelihood of the multivariate-adjusted models with and without the cross-product interaction term [24].

To assess the additive interaction between birth weight at term and unhealthy lifestyle on risk of hypertension, we considered birth weight at term and the number of unhealthy lifestyle factors as two continuous variables and assessed the main effects on incident hypertension per 1-kg lower birth weight at term (RR_g1e0_), per 1-point higher unhealthy lifestyle score (RR_g0e1_), and their joint effect (RR_g1e1_), as well as the Relative Excess Risk due to Interaction (RERI), using the equation listed below as given by VanderWeele [24–26]:

$$ \mathrm{RERI}={\mathrm{e}}^{\left(\mathrm{g}1\hbox{-} \mathrm{g}0\right)\upgamma 1+\left(\mathrm{e}1\hbox{-} \mathrm{e}0\right)\upgamma 2+\left(\mathrm{g}1\mathrm{e}1\hbox{-} \mathrm{g}0\mathrm{e}0\right)\upgamma 3}\hbox{--} {\mathrm{e}}^{\left(\mathrm{g}1\hbox{-} \mathrm{g}0\right)\upgamma 1+\left(\mathrm{g}1\hbox{-} \mathrm{g}0\right)\mathrm{e}0\upgamma 3}\hbox{--}\ {\mathrm{e}}^{\left(\mathrm{e}1\hbox{-} \mathrm{e}0\right)\upgamma 2+\left(\mathrm{e}1\hbox{-} \mathrm{e}0\right)\mathrm{g}0\upgamma 3} + 1 $$

Where, g_1_ and g_0_ mean different levels of birth weight at term while e_1_ and e_0_ mean different levels of unhealthy lifestyle.

We further proceeded with the decomposition of the joint effect, e.g. the proportions attributable to a lower term birth weight alone as [(RR_g1e0_ – 1)/ (RR_g1e1_ – 1)], unhealthy lifestyle alone as [(RR_g0e1_ – 1)/ (RR_g1e1_ – 1)], and to their interaction using the equations as [RERI/(RR_g1e1_ – 1)], the detail information on the equation has been previously published [25, 26].

We also calculated the population-attributable risk (PAR%) using the standard equation [27] as,

$$ \mathrm{P}\mathrm{A}\mathrm{R}=\left[\left(\mathrm{R}\mathrm{R}\hbox{-} 1\right),\times, \mathrm{P}\mathrm{e}\right]\div \left\{\left[\left(\mathrm{R}\mathrm{R}\hbox{-} 1\right),\times, \mathrm{P}\mathrm{e}\right]+1\right\} $$

The estimated PAR% was the percentage of incident hypertension in the study population that theoretically would not have occurred if all people had been in the low-risk group, combining a healthy birth weight at term and a healthy lifestyle, assuming a causal relation between the risk factors and hypertension.

Data were analyzed using a commercially available software program (SAS, version 9.3; SAS Institute, Inc.), and statistical significance was set at a two-tailed *P* <0.05.

## Results

Table 1 presents the age-adjusted characteristics of the participants according to the term birth weight categories. The prevalence of the lifestyle variables at baseline was similar across the categories of birth weight at term. Participants with term birth weight <2.5 or ≥4.5 kg were more likely to report a family history of hypertension. Women who had higher birth weight at term tended to have a higher adult BMI than women who were small at birth.

We documented 12,588 new cases of hypertension during the 20 years of follow-up. We observed a consistent, graded inverse association between birth weight at term and risk of hypertension (Table 2). Compared to participants in the middle category of birth weight at term (3.16–3.82 kg), the multivariate adjusted relative risk of hypertension among people with the lowest birth weight at term (<2.5 kg) was 1.25 (95 % CI, 1.14–1.37). Further adjustment for current BMI had no material impact on the association between low birth weight at term and hypertension, with a RR of 1.29 (95 % CI, 1.18–1.41).

**Table 2:** Multivariate relative risks of hypertension according to birth weight

	Term birth weight categories (kg)					*P* trend
	<2.5	2.5–3.15	3.16–3.82	3.83–4.49	≥4.5	
Cases/Person-years (PY)	510/27,436	4,077/248,172	6,330/450,509	1,541/116,911	130/11,597	
Incidence rate (per 10^5^ PY)	1,859	1,643	1,405	1,318	1,121	
Age adjusted RR (95 % CI)	1.28 (1.17–1.40)	1.17 (1.13–1.22)	1.0 (ref.)	0.96 (0.91–1.02)	0.78 (0.66–0.93)	<0.0001
Multivariable adjusted *	1.25 (1.14–1.37)	1.17 (1.12–1.21)	1.0 (ref.)	0.95 (0.90–1.01)	0.74 (0.62–0.88)	<0.0001
Further adjusted BMI **	1.29 (1.18–1.41)	1.20 (1.15–1.25)	1.0 (ref.)	0.90 (0.86–0.96)	0.67 (0.56–0.79)	<0.0001

Multivariable adjusted relative risk estimated from Cox proportional hazards models

^*^ Adjusted for age, ethnicity (Caucasian, yes/no), family history of hypertension (yes/no), use of oral contraceptive pills (never, past or current), smoking status (never smoker, former smoker, current smoker: 1–14, 15–24 or ≥25 cigarettes/d), alcohol drinking (g/d: 0, 0.1–4.9, 5.0–9.9, 10.0–14.9, 15.0–29.9, and ≥30), exercise (hours/week: 0, 0.01–1.0, 1.0–3.5, 3.5–6.0, ≥6), the DASH score (quintile), supplemental folic acid intake (no, <400, 400–800 or >800 μg/d), use of aspirin or aspirin-containing products, ibuprofen and acetaminophen (each: <1, 1, 2–3, ≥4 days/week)

^**^ Further adjusted for body mass index (kg/m^2^: <21, 21–24.9, 25–29.9, 30–31.9, ≥32)

**Fig. 1:**
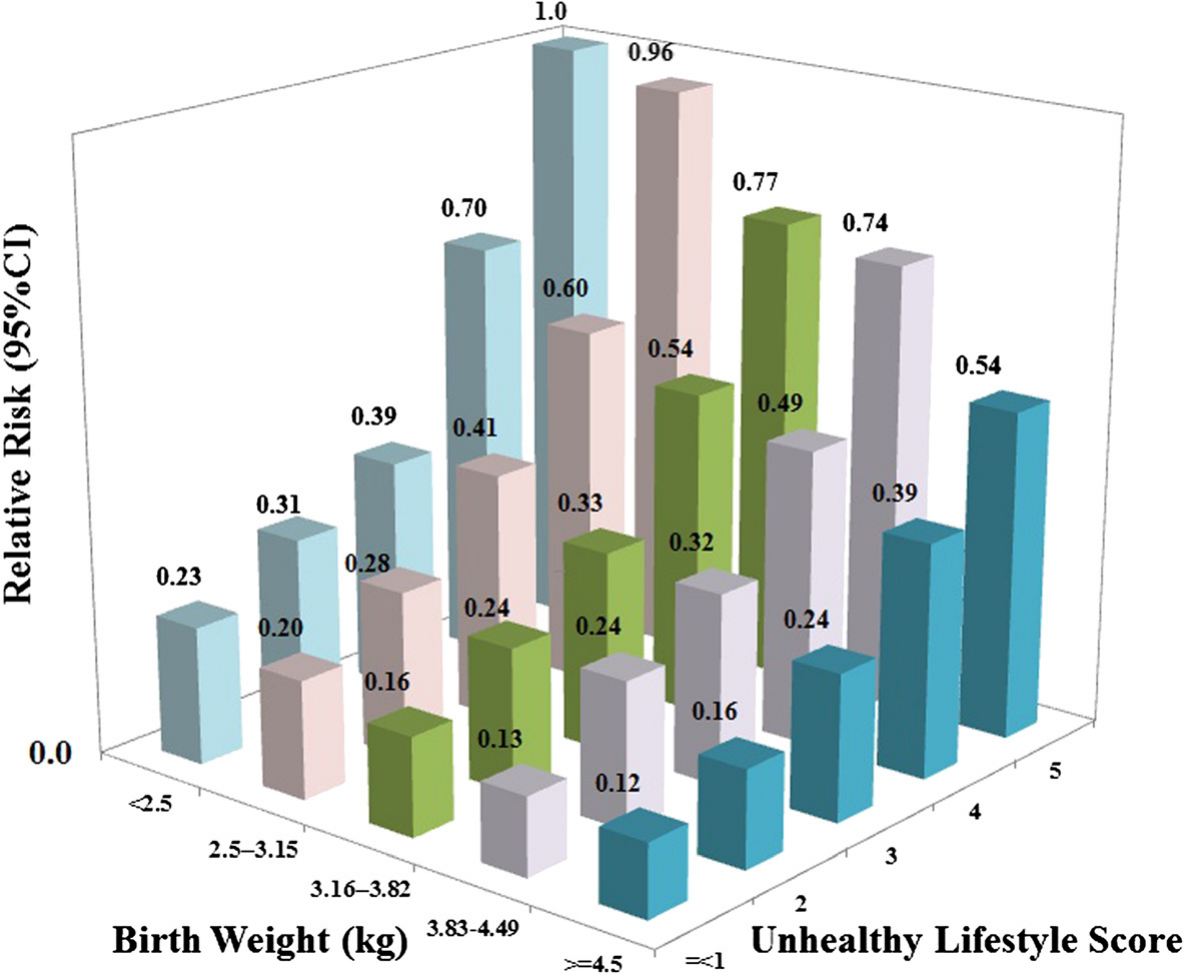
Multivariate relative risks of hypertension according to joint categories of birth weight at term and unhealthy lifestyle based on NHS2 1991–2011. Unhealthy lifestyles include exercise <3.5 hours/week at moderate intensity, diet in bottom four quintiles of the DASH score, BMI ≥25 kg/m^2^, not moderate alcohol consumption (moderate: 0.1–10 g alcohol/d), and use of non-narcotic analgesic medications at least once per week. Multivariable adjusted relative risk estimated from Cox proportional hazards models adjusted for age, ethnicity, and family history of hypertension, smoking status (never smoker, former smoker, current smoker: 1–14, 15–24, or ≥25 cigarettes/d), supplemental folic acid intake, and oral contraceptive use.

We further classified the participants according to the joint categories of birth weight at term and the unhealthy lifestyle score, and defined the group with the highest risk as the reference (birth weight at term of <2.5 kg and five unhealthy lifestyle score). The graded decreasing risk of hypertension with increasing of birth weight at term appeared consistent across all levels of unhealthy lifestyle factors (*P* for multiplicative interaction = 0.99, Fig. 1). Compared to the reference group, the multivariate-adjusted relative risk (RR) of hypertension was 0.13 (95 % CI, 0.09–0.18) among women with ≤1 unhealthy lifestyle factors combined with birth weight at term of 3.83–4.49 kg.

The RRs of hypertension were 1.23 (95 % CI, 1.11–1.36) per 1-kg lower birth weight at term and 1.61 (95 % CI, 1.51–1.71) per 1-point higher unhealthy lifestyle score. In addition, we observed that the risk of hypertension associated with a combination of low birth weight at term and unhealthy lifestyle factors (RR, 1.95; 95 % CI, 1.83–2.07) was more than addition of the risk associated with each individual factor, indicating a significant interaction on an additive scale (*P* for additive interaction <0.0001; Table 3). The proportions of the association attributable to low birth weight alone, unhealthy lifestyle alone, and their joint effect were 23.9 % (95 % CI, 16.6–31.2), 63.7 % (95 % CI, 60.4–66.9), and 12.5 % (95 % CI, 9.9–15.0), respectively (Table 3). When we stratified the analysis by participants’ age at baseline, the proportions attributable to additive interaction were 15.9 % (95 % CI, 8.9–22.9) for age ≤30 years, 12.9 % (95 % CI, 8.6–17.1) for 31–35 years, and 11.8 % (95 % CI, 8.0–15.6) for ≥36 years (Table 3).

**Table 3:** Attributing effects to additive interaction between term birth weight and lifestyle on risks of hypertension^*^

	ALL	Baseline age (years)		
		≤30	31–35	≥36
		(n = 8,652) **	(n = 17,237) **	(n = 26,315) **
Main Effects				
Lower birth weight at term (per kg)	1.23 (1.11–1.36)	1.22 (0.88–1.69)	1.25 (1.03–1.51)	1.21 (1.06–1.37)
Unhealthy lifestyle (per score) ***	1.61 (1.51–1.71)	1.73 (1.41–2.12)	1.67 (1.48–1.88)	1.55 (1.43–1.68)
Joint effect	1.95 (1.83–2.07)	2.13 (1.74–2.52)	2.05 (1.82–2.28)	1.86 (1.70–2.01)
Relative excess risk due to interaction (RERI)				
RERI	0.12 (0.09–0.15)	0.18 (0.05–0.31)	0.14 (0.07–0.20)	0.10 (0.07–0.13)
*P*	<0.0001	0.006	<0.0001	<0.0001
Attributable proportion, %				
Lower birth weight at term (per kg)	23.9 (16.6–31.2)	19.3 (–2.3–40.9)	23.7 (11.1–36.2)	24.2 (14.0–34.4)
Unhealthy lifestyle	63.7 (60.4–66.9)	64.8 (55.6–74.0)	63.5 (57.8–69.2)	64.0 (59.6–68.4)
Additive interaction	12.5 (9.9–15.0)	15.9 (8.9–22.9)	12.9 (8.6–17.1)	11.8 (8.0–15.6)

* Multivariable adjusted relative risk estimated from Cox proportional hazards models adjusted for age, ethnicity (Caucasian, yes/no), family history of hypertension (yes/no), use of oral contraceptive pills (never, past or current), smoking status (never smoker, former smoker, current smoker: 1–14, 15–24 or ≥25 cigarettes/d), and supplemental folic acid intake (no, <400, 400–800, or >800 μg/d)

** Baseline sample size

*** Unhealthy lifestyles include exercise <3.5 hours/week at moderate intensity, diet in bottom 4 quintiles of the DASH score, BMI ≥25 kg/m^2^, and not moderate alcohol consumption (moderate: 1 to 10 g alcohol/d) and use of nonnarcotic analgesic medications at least once per week

Compared to the rest of the cohort, women with birth weight at term of 2.5–4.49 kg and all the five healthy lifestyles had a relative risk of 0.33 (95 % CI, 0.26–0.43) for risk of hypertension. The PAR% for not being in this group was 66.3 %, indicating 66 % of the new cases of hypertension in our cohort could have potentially been prevented if they had all five healthy lifestyle factors combined with a birth weight at term 2.5–4.49 kg (Table 4). The PAR% for not being in the low-risk group was 76.1 % (41.5–91.4) for those ≤30 years, 63.8 % (95 % CI, 44.7–77.3) for 31–35 years, and 66.0 % (95 % CI, 53.7–75.6) for ≥36 years (Table 4). In addition, the PAR% for not being in the low-risk group was 63.3 % (95 % CI, 50.4–73.4) among women with a family history of hypertension and 70.5 % (95 % CI, 55.6–81.0) among women without a family history. In the sensitivity analysis, the women with birth weight at term of 2.5–4.49 kg and having four healthy lifestyles without considering non-narcotic analgesics was 55.5 % (95 % CI, 48.1–62.0).

**Table 4:** Multivariate relative and hypothesized population attributable risks (PARs) of incident hypertension *,**

No. of low-risk factors	Percentage of population	No. of cases of diabetes	Relative risk (95 % CI)	Population attributable risk
Total NHS II				
4: Birth weight 2.5–4.49 kg plus three healthy lifestyles				
Highest DASH quintile, daily vigorous exercise, and alcohol 0.1–10 g/d	3.93	317	0.72 (0.65–0.81)	27.0 (18.9–34.7)
5: The above four factors plus BMI <25 kg/m^2^	2.90	156	0.44 (0.37–0.51)	55.5 (48.1–62.0)
6: The above five factors plus non-narcotic analgesic use	1.58	60	0.33 (0.26–0.43)	66.3 (56.9–74.0)
Baseline age <30 years^‡^				
4: Birth weight 2.5–4.49 kg plus three healthy lifestyles				
Highest DASH quintile, daily vigorous exercise, and alcohol 0.1–10 g/d	3.45	22	0.64 (0.42–0.98)	35.1 (6.0–58.8)
5: The above four factors plus BMI <25 kg/m^2^	2.59	6	0.28 (0.14–0.56)	71.4 (45.6–86.2)
6: The above five factors plus nonnarcotic analgesic use	1.58	4	0.24 (0.09–0.63)	76.1 (41.5–91.4)
Baseline age 31–35 years^‡^				
4: Birth weight 2.5–4.49 kg plus three healthy lifestyles				
Highest DASH quintile, daily vigorous exercise, and alcohol 0.1–10 g/d	3.81	93	0.79 (0.65–0.97)	20.3 (3.8–35.6)
5: The above four factors plus BMI <25 kg/m^2^	2.91	44	0.44 (0.33–0.59)	55.5 (41.1–67.3)
6: The above five factors plus nonnarcotic analgesic use	1.64	19	0.36 (0.23–0.56)	63.6 (44.7–77.3)
Baseline age ≥36 years^‡^				
4: Birth weight 2.5–4.49 kg plus three healthy lifestyles				
Highest DASH quintile, daily vigorous exercise, and alcohol 0.1–10 g/d	4.18	202	0.70 (0.61–0.81)	29.1 (19.1–38.4)
5: The above four factors plus BMI <25 kg/m^2^	2.99	104	0.46 (0.38–0.56)	53.5 (44.0–61.8)
6: The above five factors plus nonnarcotic analgesic use	1.54	37	0.34 (0.24–0.46)	66.0 (53.7–75.6)

* Relative risks compared individuals in the low-risk category with the rest of the population; Adjusted for age (in 5-year categories), time periods, presence of a family history of hypertension, ethnicity, use of oral contraceptive pills, supplemental folic acid intake, smoking status, and the lifestyle factors that’s not included in the subgroup categories, the five lifestyle factors included nonnarcotic analgesic use, exercise, DASH score, BMI, and alcohol consumption

** The population-attributable risk is the percentage of cases of hypertension in the population that would theoretically not have occurred if all individuals had been in the low-risk category for these factors

^**‡**^Analysis stratified by age at baseline

We performed several sensitivity analyses. In order to examine potential confounding of socioeconomic status (SES), we added self-ranking of their standing in US society, including their money, education, and jobs, as a surrogate of the SES to the multivariable-adjusted model. The SES adjusted RRs of hypertension were 1.22 (95 % CI, 1.10–1.35) per 1-kg lower birth weight at term, 1.59 (95 % CI, 1.49–1.69) per 1-point higher unhealthy lifestyle score, and 1.93 (95 % CI, 1.81–2.05) for their joint effect, with a RERI of 0.12 (95 % CI, 0.09–0.14; *P* for additive interaction <0.0001). When we applied the expanded low-risk lifestyle score, the RRs of hypertension were 1.28 (95 % CI, 1.17–1.40) per 1-kg lower birth weight at term, 1.46 (95 % CI, 1.40–1.51) per 1-point higher unhealthy lifestyle score (5- expanded low-risk lifestyle score), and 1.84 (95 % CI, 1.74–1.94) for their joint effect, with a RERI of 0.10 (95 % CI, 0.08–0.13; *P* for additive interaction <0.0001). We also performed analysis of the main research question based on the cross-sectional data at baseline including all participants without missing data of birth weight and lifestyle factors. The odds ratios of hypertension were 1.09 (95 % CI, 0.93–1.28) per 1-kg lower birth weight at term, 1.62 (95 % CI, 1.51–1.75) per 1-point higher unhealthy lifestyle score, and 1.78 (95 % CI, 1.60–1.97) for their joint effect, with a RERI of 0.07 (95 % CI, 0.02–0.13; *P* for additive interaction of 0.005).

## Discussion

After 20 years of follow-up of a large population of initially hypertension-free young women, we observed that the risk of hypertension associated with a combination of low birth weight at term and unhealthy lifestyle factors was more than the addition of the risk associated with each of these factors, indicating a significant interaction on an additive scale. Our data indicate that combination of a healthy birth weight and a healthy adulthood lifestyle could prevent 66 % of the cases of hypertension in this population.

It is hypothesized that restricted fetal growth reflects maternal vascular stress inherited by her children, and which may also interact with adulthood lifestyle [28, 29]. Even though the precise mechanisms remain unclear, previous data have suggested that a reduced number of nephrons associated with low birth weight might play a pivotal role [30, 31]. A reduced nephron number may lead to overworking or hyper-filtration of glomeruli [32], and exacerbate the effects of other risk factors, such as a high salt intake, on hypertension [33, 34]. Other factors involved in the developmental programming of hypertension include vascular structural and functional changes [35], neuroendocrine adaptations to stress, insulin sensitivity, and sympathetic nervous system activity [36, 37]. To our knowledge, our study provides, for the first time, evidence that fetal growth restriction may interact with later lifestyle to increase adulthood hypertension risk. This finding is in line with previous observations. For example, adult blood pressure was more markedly affected by obesity among individuals with low birth weight [38] than normal birth weight individuals or among individuals exposed to famine prenatally than non-exposed individuals [10]. In our earlier analyses in a large Chinese cohort [8], we found that the associations of fetal famine exposure cohort with increased blood pressure in adulthood appeared to be stronger among adults who were overweight or had a Western dietary pattern in later life. Our finding in the current study provided further evidence that individuals with fetal growth restriction may be more sensitive to the adverse effects of later life unhealthy lifestyles on hypertension risk.

It is widely accepted that hypertension can be prevented by lifestyle modifications. The National High Blood Pressure Education Program Coordinating Committee published its first statement on the primary prevention of hypertension in 1993 [39] and updated it in 2002 [13]. The recommended lifestyle modifications for primary prevention of hypertension include maintaining a normal body weight, dietary modifications, engaging in regular aerobic physical activity, and limiting alcohol consumption [13]. Recent meta-analyses of lifestyle-related intervention studies provided solid evidence for the primary prevention of hypertension by modifications of those lifestyle factors [40, 41]. In a previous study of NHS II between 1991 and 2005 [12], a hypothetical PAR of 78 % was observed for women who lacked the six low-risk lifestyle factors, including the five low-risk lifestyle factors included in the current analysis and intake of 400 μg/d or more of supplemental folic acid [12]. Compared to the previous analysis, we did not include supplemental folic acid as a low-risk lifestyle factor as the nationwide fortification of enriched uncooked cereal grains with folic acid in the United States became mandatory from 1998 [42]; after that, the mean serum folate level has stayed at a much higher level [43], and thus the benefit of further supplementation of folic acid is not clear. In the current study, the observed PAR% for the absence of six low-risk factors, including healthy birth weight and five low-risk lifestyle factors, was 66 %, which is somewhat lower than the previous study [12]. One reason for such difference is that the current cohort included six additional years of follow-up and thus the participants became much older. When we stratified the study population by baseline age, the PAR% for absence of five low-risk factors and birth weight within 2.5–4.49 kg was 76 % among women who were less than 30 years of age at baseline, which was higher than the previous estimation of the five low-risk factors (72 %) [12]. The difference of PAR% across different age groups is consistent with findings from our previous observation [44] and highlights the importance of lifestyle modifications at early age.

Our study also indicates that 12 % of hypertension cases may only occur if both unhealthy birth weight and unhealthy lifestyle were present, not if only one or the other is present [24]. This finding is important not only for the primary prevention of hypertension, but also for understanding the mechanism [45]. Specifically, a percentage of hypertension cases appeared to be related to the additive effects of both prenatal and later life factors, providing new evidence in this research area [28, 29]. As low birth weight itself is not a causal factor in the fetal programming of adult disease but an indicator of intra-uterine adversity that increases the risk of hypertension in adulthood, our findings emphasize the importance of prevention of fetal growth restriction that may be due to modifiable risk factors, such as maternal nutrition and smoking [46, 47]. Adoption of a healthy lifestyle by young women could not only benefit them, but also prevent hypertension in their offspring [48].

A major strength of the present study is decomposition of the joint effect of birth weight and adulthood lifestyle factors. Our study, for the first time, quantitatively estimated the joint effects of prenatal and adulthood risk factors on risk of hypertension. Other strengths of the present study include the large number of incident hypertension cases, long-term follow-up, and repeated measurement of lifestyle factors during the 20 years of follow-up.

Our study has several limitations. First, our cohorts included mostly Caucasian women and the PAR was population-specific, which limited the generalizability to men or other ethnic groups of women. However, the relative homogeneity of the study populations in educational attainment and SES enhances the internal validity. The prevalence of low-risk factors in the NHS II women is much higher than that among Black and Hispanic women [49], while the percentage of low birth weight [50] is greater in the general US population than that in our cohorts. Therefore, the impact of unhealthy lifestyle and low birth weight would be greater in more racially diverse populations. Second, we could not exclude the possibility of exposure misclassification of the questionnaire-based assessment of lifestyle factors. However, the prospective study design indicates such bias would likely be random with respect to outcome status, resulting in attenuation of the effect estimates, thus underestimating the true associations. This study was also limited by its reliance on self-reported birth weight and lifestyle factors. As discussed previously [14, 51, 52], missing birth weight or lifestyle factors was likely to be random in our cohort, and therefore unlikely to affect the associations we observed artefactually. Although we have adjusted for family history of hypertension, residual confounding from genetic effect still could not be totally ruled out. Recent genome-wide association meta-analysis identified seven loci associated with birth weight, and one of these (ADRB1) was also associated with adult blood pressure [53]. Unmeasured confounding might also exist even though we have controlled for a wide range of risk factors for hypertension. However, only a very strong unmeasured risk factor for hypertension together with a very large prevalence imbalance among exposure groups could explain our findings [54, 55].

## Conclusion

In conclusion, our findings suggest that the effects of fetal growth restriction and unhealthy lifestyle on the risk of hypertension are greater than additive. Though some cases of hypertension may only be prevented by simultaneous improvement of both prenatal and postnatal factors, the majority of cases of hypertension could be prevented by the adoption of a healthier lifestyle.

## Abbreviations

BMI: Body mass index
CI: Confidence interval
DASH: Dietary Approaches to Stop Hypertension
FFQ: Food Frequency Questionnaire
NHS II: Nurses’ Health Study II
PAR%: Population-attributable risk
RR: Relative risk
SES: Socioeconomic status

## References

[1] Nwankwo T, Yoon SS, Burt V, Gu Q. Hypertension among adults in the United States: National Health and Nutrition Examination Survey, 2011–2012. NCHS data brief 2013, p. 1–8. http://www.cdc.gov/nchs/data/databriefs/db133.pdf. 24171916

[2] Lim SS, Vos T, Flaxman AD, Danaei G, Shibuya K, Adair-Rohani H (2012). A comparative risk assessment of burden of disease and injury attributable to 67 risk factors and risk factor clusters in 21 regions, 1990–2010: a systematic analysis for the Global Burden of Disease Study 2010. Lancet.

[3] Danaei G, Ding EL, Mozaffarian D, Taylor B, Rehm J, Murray CJ (2009). The preventable causes of death in the United States: comparative risk assessment of dietary, lifestyle, and metabolic risk factors. PLoS Med.

[4] Huxley R, Neil A, Collins R (2002). Unravelling the fetal origins hypothesis: is there really an inverse association between birthweight and subsequent blood pressure?. Lancet.

[5] Huxley RR, Shiell AW, Law CM (2000). The role of size at birth and postnatal catch-up growth in determining systolic blood pressure: a systematic review of the literature. J Hypertens.

[6] Law CM, Shiell AW (1996). Is blood pressure inversely related to birth weight? The strength of evidence from a systematic review of the literature. J Hypertens.

[7] Li Y, He Y, Qi L, Jaddoe VW, Feskens EJ, Yang X (2010). Exposure to the Chinese famine in early life and the risk of hyperglycemia and type 2 diabetes in adulthood. Diabetes.

[8] Li Y, Jaddoe VW, Qi L, He Y, Lai J, Wang J (2011). Exposure to the Chinese famine in early life and the risk of hypertension in adulthood. J Hypertens.

[9] Li Y, Jaddoe VW, Qi L, He Y, Wang D, Lai J (2011). Exposure to the Chinese famine in early life and the risk of metabolic syndrome in adulthood. Diabetes Care.

[10] Stanner SA, Bulmer K, Andres C, Lantseva OE, Borodina V, Poteen VV (1997). Does malnutrition in utero determine diabetes and coronary heart disease in adulthood? Results from the Leningrad siege study, a cross sectional study. BMJ.

[11] Stein AD, Zybert PA, van der Pal-de Bruin K, Lumey LH (2006). Exposure to famine during gestation, size at birth, and blood pressure at age 59 y: evidence from the Dutch Famine. Eur J Epidemiol.

[12] Forman JP, Stampfer MJ, Curhan GC (2009). Diet and lifestyle risk factors associated with incident hypertension in women. JAMA.

[13] Whelton PK, He J, Appel LJ, Cutler JA, Havas S, Kotchen TA (2002). Primary prevention of hypertension: clinical and public health advisory from The National High Blood Pressure Education Program. JAMA.

[14] Curhan GC, Chertow GM, Willett WC, Spiegelman D, Colditz GA, Manson JE (1996). Birth weight and adult hypertension and obesity in women. Circulation.

[15] Forman JP, Curhan GC, Taylor EN (2008). Plasma 25-hydroxyvitamin D levels and risk of incident hypertension among young women. Hypertension.

[16] Colditz GA, Martin P, Stampfer MJ, Willett WC, Sampson L, Rosner B (1986). Validation of questionnaire information on risk factors and disease outcomes in a prospective cohort study of women. Am J Epidemiol.

[17] Troy LM, Michels KB, Hunter DJ, Spiegelman D, Manson JE, Colditz GA (1996). Self-reported birthweight and history of having been breastfed among younger women: an assessment of validity. Int J Epidemiol.

[18] Appel LJ, Moore TJ, Obarzanek E, Vollmer WM, Svetkey LP, Sacks FM (1997). A clinical trial of the effects of dietary patterns on blood pressure. DASH Collaborative Research Group. NEJM.

[19] Dauchet L, Kesse-Guyot E, Czernichow S, Bertrais S, Estaquio C, Péneau S (2007). Dietary patterns and blood pressure change over 5-y follow-up in the SU.VI.MAX cohort. Am J Clin Nutr.

[20] Curhan GC, Willett WC, Rosner B, Stampfer MJ (2002). Frequency of analgesic use and risk of hypertension in younger women. Arch Intern Med.

[21] Dedier J, Stampfer MJ, Hankinson SE, Willett WC, Speizer FE, Curhan GC (2002). Nonnarcotic analgesic use and the risk of hypertension in US women. Hypertension.

[22] Forman JP, Stampfer MJ, Curhan GC (2005). Non-narcotic analgesic dose and risk of incident hypertension in US women. Hypertension.

[23] Li R, Chambless L (2007). Test for additive interaction in proportional hazards models. Ann Epidemiol.

[24] VanderWeele TJ, Asomaning K, Tchetgen Tchetgen EJ, Han Y, Spitz MR, Shete S (2012). Genetic variants on 15q25.1, smoking, and lung cancer: an assessment of mediation and interaction. Am J Epidemiol.

[25] VanderWeele TJ, Tchetgen EJ (2014). Attributing effects to interactions. Epidemiology.

[26] VanderWeele TJ, Knol MJ. A tutorial on interaction. Epidemiol. Methods 2014;3:33–72.

[27] Wacholder S, Benichou J, Heineman EF, Hartge P, Hoover RN (1994). Attributable risk: advantages of a broad definition of exposure. Am J Epidemiol.

[28] Barker DJ (1995). Fetal origins of coronary heart disease. BMJ.

[29] Gluckman PD, Hanson MA, Cooper C, Thornburg KL (2008). Effect of in utero and early-life conditions on adult health and disease. N Engl J Med.

[30] Bauer R, Walter B, Bauer K, Klupsch R, Patt S, Zwiener U (2002). Intrauterine growth restriction reduces nephron number and renal excretory function in newborn piglets. Acta Physiol Scand.

[31] Hughson MD, Douglas-Denton R, Bertram JF, Hoy WE (2006). Hypertension, glomerular number, and birth weight in African Americans and white subjects in the southeastern United States. Kidney Int.

[32] Keller G, Zimmer G, Mall G, Ritz E, Amann K (2003). Nephron number in patients with primary hypertension. N Engl J Med.

[33] Nehiri T, Duong Van Huyen JP, Viltard M, Fassot C, Heudes D, Freund N (2008). Exposure to maternal diabetes induces salt-sensitive hypertension and impairs renal function in adult rat offspring. Diabetes.

[34] Salazar F, Reverte V, Saez F, Loria A, Llinas MT, Salazar FJ (2008). Age- and sodium-sensitive hypertension and sex-dependent renal changes in rats with a reduced nephron number. Hypertension.

[35] Karatza AA, Varvarigou A, Lumey LH, Vaiserman A (2013). Intrauterine growth restriction and the developing vascular tree. Early life nutrition and adult health and development.

[36] Baum M (2010). Role of the kidney in the prenatal and early postnatal programming of hypertension. Am J Physiol Renal Physiol.

[37] Luyckx VA, Bertram JF, Brenner BM, Fall C, Hoy WE, Ozanne SE (2013). Effect of fetal and child health on kidney development and long-term risk of hypertension and kidney disease. Lancet.

[38] Leon DA, Koupilova I, Lithell HO, Berglund L, Mohsen R, Vagero D (1996). Failure to realise growth potential in utero and adult obesity in relation to blood pressure in 50 year old Swedish men. BMJ.

[39] National High Blood Pressure Education Program Working Group report on primary prevention of hypertension. Arch Internal Med. 1993;153:186–208. 8422207

[40] Baena CP, Olandoski M, Younge JO, Buitrago-Lopez A, Darweesh SK, Campos N (2014). Effects of lifestyle-related interventions on blood pressure in low and middle-income countries: systematic review and meta-analysis. J Hypertens.

[41] Dickinson HO, Mason JM, Nicolson DJ, Campbell F, Beyer FR, Cook JV (2006). Lifestyle interventions to reduce raised blood pressure: a systematic review of randomized controlled trials. J Hypertens.

[42] U.S. Food and Drug Administration. Food standards: amendment of standards of identity for enriched grain products to require addition of folic acid. Final rule. 21 CFR Parts 136, 137, and 139. Fed Regist 1996. http://www.gpo.gov/fdsys/pkg/FR-1996-09-05/pdf/96-22606.pdf.

[43] Pfeiffer CM, Hughes JP, Lacher DA, Bailey RL, Berry RJ, Zhang M (2012). Estimation of trends in serum and RBC folate in the U.S. population from pre- to postfortification using assay-adjusted data from the NHANES 1988–2010. J Nutr.

[44] Cohen L, Curhan GC, Forman JP (2012). Influence of age on the association between lifestyle factors and risk of hypertension. J Am Soc Hypertens.

[45] VanderWeele TJ, Robins JM (2007). The identification of synergism in the sufficient-component-cause framework. Epidemiol.

[46] Persson LA, Arifeen S, Ekstrom EC, Rasmussen KM, Frongillo EA, Yunus M (2012). Effects of prenatal micronutrient and early food supplementation on maternal hemoglobin, birth weight, and infant mortality among children in Bangladesh: the MINIMat randomized trial. JAMA.

[47] Ko TJ, Tsai LY, Chu LC, Yeh SJ, Leung C, Chen CY (2014). Parental smoking during pregnancy and its association with low birth weight, small for gestational age, and preterm birth offspring: a birth cohort study. Pediatr Neonatol.

[48] de Jonge LL, Harris HR, Rich-Edwards JW, Willett WC, Forman MR, Jaddoe VW (2013). Parental smoking in pregnancy and the risks of adult-onset hypertension. Hypertension.

[49] Schoenborn CA, Adams PF. Health behaviors of adults: United States, 2005–2007. Vital Health Stat 10. 2010;245:1–132. 20669609

[50] U.S. Department of Health and Human Services, Health Resources and Services Administration, Maternal and Child Health Bureau. Child Health USA 2013. Rockville, Maryland: U.S. Department of Health and Human Services, 2013. http://mchb.hrsa.gov/publications/pdfs/childhealth2013.pdf. Accessed 23 May 2015.

[51] Rich-Edwards JW, Colditz GA, Stampfer MJ, Willett WC, Gillman MW, Hennekens CH (1999). Birthweight and the risk for type 2 diabetes mellitus in adult women. Ann Intern Med.

[52] Rich-Edwards JW, Kleinman K, Michels KB, Stampfer MJ, Manson JE, Rexrode KM (2005). Longitudinal study of birth weight and adult body mass index in predicting risk of coronary heart disease and stroke in women. BMJ.

[53] Horikoshi M, Yaghootkar H, Mook-Kanamori DO, Sovio U, Taal HR, Hennig BJ (2013). New loci associated with birth weight identify genetic links between intrauterine growth and adult height and metabolism. Nat Genet.

[54] Schneeweiss S (2006). Sensitivity analysis and external adjustment for unmeasured confounders in epidemiologic database studies of therapeutics. Pharmacoepidemiol Drug Saf.

[55] Lee WC (2011). Bounding the bias of unmeasured factors with confounding and effect-modifying potentials. Stat Med.

